# *In vitro *cytotoxicity of two novel oral formulations of Amphotericin B (iCo-009 and iCo-010) against *Candida albicans*, human monocytic and kidney cell lines

**DOI:** 10.1186/1476-511X-10-144

**Published:** 2011-08-20

**Authors:** Carlos G Leon, Jinkyung Lee, Karen Bartlett, Pavel Gershkovich, Ellen K Wasan, Jinying Zhao, John G Clement, Kishor M Wasan

**Affiliations:** 1 Division of Pharmaceutics and Biopharmaceutics, Faculty of Pharmaceutical Sciences, The University of British Columbia, Vancouver British Columbia, V6T 1Z3, Canada; 2 Faculty of Environmental and Occupational Health, The University of British Columbia, Vancouver, British Columbia, V6T 1Z3, Canada; 3 School of Health Sciences, British Columbia Institute of Technology, 3700 Willingdon Avenue, Burnaby, British Columbia, V5G 3H2, Canada; 4 iCo Therapeutics Inc., 760-777 Hornby, Vancouver, British Columbia, V6Z 1S4, Canada

**Keywords:** Amphotericin B, Candida, cytotoxicity, monocytes.

## Abstract

**Background:**

Invasive fungal infections such as candidiasis constitute an increasingly important medical problem. Drugs currently used for the treatment of candidiasis include polyenes (such as Amphotericin B) and azoles. Amphotericin B (AmpB) presents several limitations such as its nephrotoxicity and limited solubility. We have developed two novel lipid-based AmpB formulations which *in vivo *show less nephrotoxicity and enhanced solubility compared to Fungizone™ a commercial AmpB formulation.

The purpose of this study was to determine the cytotoxicity of Fungizone™, Ambisome™ and two novel AmpB formulations (iCo-009 and iCo-010) against *Candida albicans*, human kidney (293T) cells and monocytic (THP1) cells.

**Methods:**

Cell cytotoxicity to the AmpB formulations was evaluated by MTS and LDH assays. *In vitro *anti-*Candida albicans *activity was assessed after a 48 h drug incubation.

**Results:**

None of the AmpB formulations tested showed cytotoxicity against 293T cells. In the case of THP1 cells only Fungizone™ and Ambisome™ showed cytotoxicity at 500 μg/L (n = 4-10, p < 0.05).

The calculated EC50 to *Candida albicans *for the different formulations was as follows: 26.8 ± 2.9 for iCo-010, 74.6 ± 8.9 for iCo-009, 109 ± 31 for Ambisome™ and 87.1 ± 22 for Fungizone™ (μg of AmpB/L, n = 6-12, p < 0.05).

**Conclusions:**

The AmpB formulations analyzed were not cytotoxic to 293T cells. Cytotoxicity in THP1 cells was observed for Fungizone™ and Ambisome™, but not with the novel AmpB formulations. iCo-010 had higher efficacy compared to other three AmpB formulations in the *Candida albicans *model.

The absence of cytotoxicity as well as its higher efficacy for the *Candida *model compared to Fungizone™ and Ambisome™ suggest that iCo-010 has potential in treating candidiasis.

## 1. Background

Invasive fungal infections constitute an increasingly important medical problem due to the growth of immunodeficient populations, the development of antifungal resistance and limitations in the efficacy and toxicity of current antifungals [1]. Candida species are the most common cause of nosocomial invasive mycosis and are the leading cause of related mortality [2]. An increasing rate of candidaemia [3] as well as the emergence of drug resistant strains [4] support the efforts in discovering novel therapeutic approaches [1].

Drugs currently used for the treatment of candidiasis include polyenes (such as Amphotericin B AmpB), azoles (fluconazole), echinocandins and flucytosine. Limited therapeutic efficiency and drug resistance have led to consider new therapeutic approaches, in particular the use of new analogs of existing drugs [1].

Amphotericin B (AmpB) is a treatment of choice for systemic fungal infections [5]. The mechanism of action of AmpB involves its binding to ergosterol in the fungal cell membrane, producing pores in the membrane which leads to ion loss and cell death [6]. Recently it has been described that AmpB can also elicit cell death through the induction of a strong oxidative burst [7]. Acquired resistance to Amphotericin B in Candida species, though rare, has been reported previously [8,9]. This AmpB resistance has been correlated with decreased levels of ergosterol in the plasma membrane and has been accompanied with azole resistance [10,11].

One of the major limitations associated with AmpB is its nephrotoxicity [12]. Another issue related to AmpB is its poor solubility that limits its route of administration [13]. The development of an effective, safe, and inexpensive oral formulation of amphotericin B would have many applications for the treatment of fungal diseases. The new oral amphotericin B formulations developed by our group, iCo-009 and iCo-010, show efficacy *in vivo *against leishmaniasis [14,15], aspergillosis and candidiasis [16].

The purpose of the present study was to determine if four different formulations of AmpB (iCo-009, iCo-010, Fungizone™ and Ambisome™) were cytotoxic in human monocytic, human kidney cells and *Candida albicans*.

## 2. Results and Discussion

**Figure 1:**
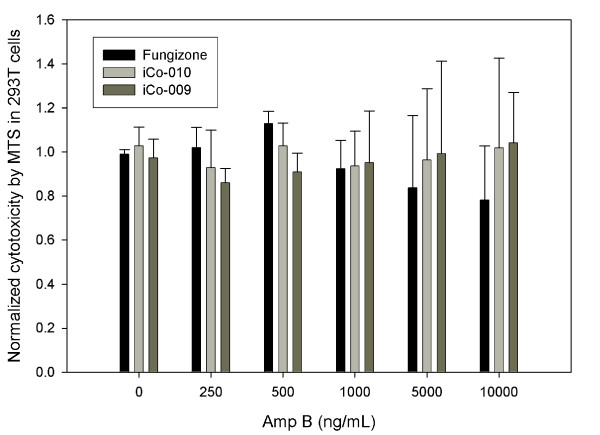
**Cytotoxicity in 293T cells**: Dose-response effects of Fungizone™, iCo-009 and iCo-010 on cytotoxicity on human embryonic (293T) kidney cells. The cells were exposed for 48 h with different drug concentrations (0, 250, 500, 1000, 5000 and 10,000 μg AmpB/L). MTS values were corrected for protein determination and normalized to the control cells. Data are reported as means ± standard deviation of four experiments (eight replicates per experiment).

**Figure 2:**
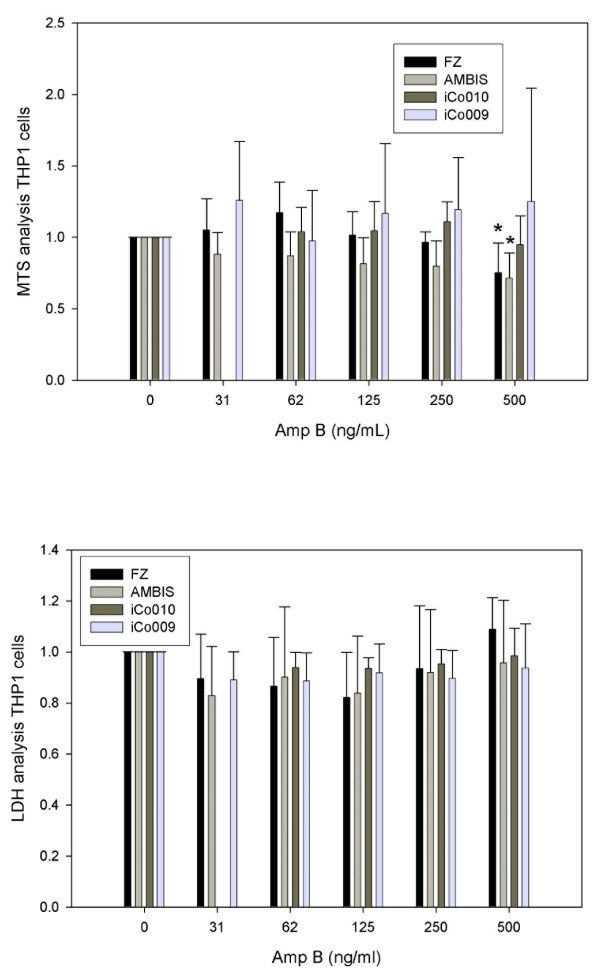
**Dose-response effects of Fungizone™, Ambisome™, iCo-009 and iCo-010 on cytotoxicity in human monocytic (THP1) cells as assessed using an MTS assay (Figure 2a)**. The cells were exposed for 48 h with different drug concentrations (31.25, 62.5, 125, 250 and 500 μg AmpB/L). The cells were washed and incubated with the MTS reagent for 90 min. Data are reported as means ± standard deviation of four to ten experiments, * p < 0.05, eight replicates per experiment). Dose-response effects of Fungizone™, Ambisome™, iCo-009 and iCo-010 on cytotoxicity in human monocytic (THP1) cells as assessed using an LDH assay **(Figure 2b)**. The cells were exposed for 48 h with different drug concentrations (31.25, 62.5, 125, 250 and 500 μg AmpB/L). An aliquot of the medium was incubated with the LDH reagent. Data are reported as means ± standard deviation of six experiments (four replicates per experiment).

The first objective of our study was to determine the AmpB-induced cytotoxicity in two human cell lines, 293T kidney and THP1 monocytic cells. No evidence of cytotoxicity was found in any of the three AmpB formulations used (Fungizone™, iCo-009 and iCo-010) at the doses tested (Figure 1, n = 4) using a respiration assay (MTS) in the kidney 293T cells. The vehicle controls for both iCo-009 and iCo-010 did not show a toxic effect (data not shown). At the highest AmpB dose analyzed (10,000 μg/L), there was a reduction of 20% in the respiration rate of the cells treated with Fungizone™, however, it didn't reach statistical significance. In terms of cytotoxicity in THP1 monocytic cells of the four AmpB formulations at the doses analyzed (0 to 500 μg/L), differences were shown between the MTS (Figure 2a, n = 4-10) and LDH (Figure 2b, n = 6) results. No changes in viability were found with the LDH assay at any of the AmpB concentrations analyzed. The LDH results were corrected by the amount of protein in each treatment group. There was also no difference in protein content in the different treatment groups. However, the Normalized MTS results exhibited a reduced respiration rate at 500 μg/L for both Fungizone™ (0.71 ± 0.04) and Ambisome™ (0.72 ± 0.17) compared to controls (1.00).

Previously, others [17] have described a difference in the degree of cellular activation by AmpB in the 293 and THP1 cell lines. This disparity was accounted by the differential expression of the TLR2, highly expressed in THP1 cells and expressed at very low levels in 293 cells. The new oral formulations, iCo-009 and iCo-010, did not show any evidence of toxicity at the highest concentration analyzed in 293T cells (10,000 μg/L, Figure 1). The limited TLR2 expression in 293T cells may explain this lack of cytotoxicity that was observed even at large doses of AmpB compared with a cytotoxic effect of AmpB in THP1 cells at a lower concentration (500 μg/L).

The difference in cytotoxicity found in THP1 cells between the LDH and MTS assays could be accounted by the fact that the MTS assay relies on the accessibility of the substrate to the respiratory machinery of the cell (i.e. expression and activity of transporters). In the case of LDH there is no active transport factor(s) as it is an index of the cell breakdown and the release of LDH enzyme to the medium. The drug concentration that elicits toxicity in the THP1 monocytes with Fungizone™ and Ambisome™ can be compared with the serum drug levels obtained in patients. After a standard dose of AmpB (0.3 mg/kg on days 1 and 2 followed by increasing doses to 0.5 mg/kg) for patient therapy, the expected serum AmpB levels can reach 200 μg/L [18]. Few studies have assessed the AmpB-induced cytotoxicity in monocytes and macrophages. Sesana *et al*., [19] examined the *in vitro *activity of AmpB cochleates against *Leishmania chagasi *and the cytotoxic effect on mouse peritoneal macrophages using AmpB deoxycholate (Fungizone™) as a control. They found that the AmpB deoxycholate (Fungizone™) was cytotoxic to mouse peritoneal macrophages at a concentration of 1250 μg/L, while the AmpB cochleates were not toxic at that AmpB concentration.

**Figure 3:**
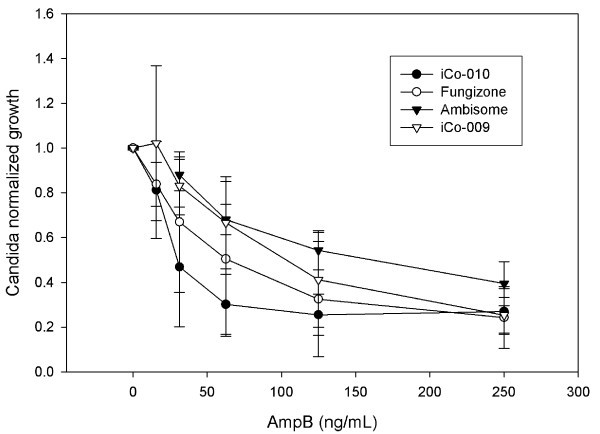
***In vitro Candida *efficacy studies**. *Candida *was exposed for 48 h to different doses of Fungizone™, iCo-009 and iCo-010 (0, 15.6, 31.25, 62.5, 125 and 250 μg AmpB/L). A vehicle control with the same lipid composition as iCo-010 was also used at comparable volumes. Growth was normalized by the corresponding absorbance of the untreated *Candida*. The vehicle control showed no effect on Candida growth. (* p < 0.05 n = 6-12 independent experiments, each experiment includes eight replicates).

The second objective of our study was to determine the efficacy of the novel AmpB formulations in an *in vitro Candida albicans *model. Unfortunately, frequently there is limited correlation between *in vitro *susceptibility determination and patient response [20]. However, use of RPMI medium supplemented with dextrose showed improvement in assessing *Candida albicans *antibiotic susceptibility [21]. Thus this methodology was used in the present study. The results (Figure 3 and Table 1) showed that iCo-010 had a lower EC50 than Fungizone™, Ambisome™ and iCo-009 for this ATCC *Candida albicans *reference strain (n = 6-12 experiments). The EC50 calculated for AmpB (as Fungizone™) for the *Candida albicans *reference strain 18804 that was assessed using this system is similar to that previously obtained for this strain in an Etest [22].

**Table 1:** EC50 for AmpB formulations (mean ± SD, expressed in **μ**g/L of AmpB, n = 6-12, * p < 0.05) tested in the *Candida *model

	Mean ± SD
Fungizone ™	87.1 ± 22

Ambisome™	109 ± 31

iCo-009	74.6 ± 8.9

iCo-010	26.8 ± 2.9 *

iCo-009 was previously compared to Abelcet^® ^in an *in vivo *study in *Candida albicans*-infected male rats [16]. In that report, iCo-009 exhibited significant reduction in viable colony forming units of *Candida *from kidney homogenates compared to Abelcet^®^.

## 3. Conclusions

We have tested the cytotoxicity and efficacy of four AmpB formulations in two human cell lines and a *Candida albicans *model, respectively. Fungizone™ and Ambisome™, but not iCo-009 and iCo-010, show cytotoxicity in human monocytes at a concentration of 500 μg/L. iCo-010 shows a higher efficacy compared to iCo-009, Ambisome™ and Fungizone™ in the *Candida albicans *model. The lack of toxicity and efficacy of iCo-010, a novel oral AmpB formulation, warrant future *in vivo Candida albicans *studies to determine its therapeutic index.

## 4. Materials and methods

### 4.1. Novel lipid oral Amphotericin B formulations

The description of the composition and development of the two novel lipid oral Amphotericin B formulations can be found elsewhere iCo-009 [16] and iCo-010 [15]. AmpB concentration was analyzed by HPLC as previously described [16]. For the cytotoxicity and efficacy studies we used as negative controls medium alone and medium containg the vehicle controls for both iCo-009 and iCo-010. No cytotoxicity was found when any of these controls were used (data not shown).

### 4.2. Cytotoxicity assay on 293T cells

Human embryonic kidney cells (293T ATCC CRL 11268) were kindly provided by Dr JS Hill (St Paul's Hospital, UBC, Vancouver, BC). Cells were grown in complete DMEM without phenol red at 37°C (5% CO_2_) in T75 flasks. At near confluency, the cells were trypsinized and transferred to either additional flasks or to 96-well plates (previously coated with Poly L-lysine and seeded at a density of 10,000 cells/well). Twenty four hours post seeding, the adherent cells were washed and incubated with different concentrations of AmpB formulations (250 to 10,000 μg/L of AmpB) for 48 h. Percent AmpB-mediated cytotoxicity was determined using the MTS conversion assay (Promega Corp, Madison, WI), as previously described [23].

### 4.3. Cytotoxicity assay on THP1 cells

Human monocytic THP1 cells (ATCC TIB-202) were kindly provided by Dr JS Hill (St Paul's Hospital, UBC, Vancouver, BC). Cells were grown in complete RPMI without phenol red at 37°C (5% CO_2_) in T75 flasks. Before the cells reached a concentration of one million per mL, the cells were transferred to either additional flasks or to 96-well plates and seeded at a density of 20,000 cells/well with the addition of PMA (10 ng/mL final) to allow the cells to differentiate overnight. Twenty four hours post seeding, the differentiated cells were washed and the different concentrations of AmpB formulations were added (31 to 500 μg/L of AmpB) for 48 h. After a 90 min incubation with the MTS reagent, percent AmpB-mediated cytotoxicity was determined using the MTS conversion assay (Promega Corp, Madison, WI), as previously described [23] or the LDH assay (Promega Corp, Madison, WI) as outlined by the manufacturer specifications. The MTS and LDH results were corrected by the amount of protein present in the respective wells. Purified LDH from the manufacturer kit was used as a positive control (data not shown).

### 4.4. Minimum inhibitory concentration determination of the AmpB formulations on *Candida *growth in liquid medium

RPMI without phenol red and supplemented with 10% Fetal bovine serum, 2% dextrose plus penicillin and streptomycin was used to prepare dilutions of AmpB (31.25, 62.5, 125, 250 and 500 μg/L- final drug concentration) from Fungizone™, Ambisome™, iCo-009 and iCo-010 (AmpB concentration in the formulations was determined by HPLC). A vehicle control of iCo-009 and iCo-010 was used as a negative control. RPMI is the standard medium used in microplate drug susceptibility assays and is commonly used for testing of drug sensitivity for both *Candida albicans *and *Cryptococcus neoformans *[24]. A limitation of the RPMI medium is that there are frequent overlaps between the EC50 ranges for isolates that are putatively resistant or susceptible to Amphotericin B. The supplementation of RPMI medium with 2% glucose has lead to higher fungal growth rates and better separation of EC50s for isolates that are putatively resistant to fluconazole [21]. This is the methodology that we have used for our experiments.

The *Candida albicans *(ATCC reference strain 18804) inoculum was prepared as follows. The fungal cell concentration was determined using a hematocytometer. One hundred microlitres of a 5 × 10^5^/mL preparation of *Candida *in RPMI was placed in the 96 well plates for a final concentration of 2.5 × 10^5 ^cells/mL. Each concentration was analyzed in eight replicates per experiment (n = 6-12 independent experiments).

The plates were incubated for 48 h at 37°C protected from the light before analyzing the growth pattern using a spectrophotometer at 650 nm. A new *Candida *agar plate was streaked each week to ensure the viability of the culture.

## Competing interests

J. G. C. is an employee/co-founder/shareholder and director of iCo Therapeutics Inc. All other authors: none to declare.

## Authors' contributions

CGL carried out the 293T cytotoxicity assays and drafted the manuscript. JL carried out the THP1 cytotoxicity assays and the Candida efficacy experiments. KB participated in the design of the study. PG prepared the AmpB formulations and edited the manuscript. EKW designed and prepared the AmpB formulations. JZ prepared and characterized the novel AmpB formulations. JGC edited the manuscript. KMW participated in the design of the study and helped to draft the manuscript. All authors read and approved the final manuscript.

## References

[B1] Calugi C Trabocchi A Guarna A Novel small molecules for the treatment of infections caused by Candida albicans: a patent review 2 Expert Opin Ther Pat 2011 21 3 381 97 10.1517/13543776.2011.551116 21241212

[B2] Marr KA Fungal infections in oncology patients: update on epidemiology, prevention, and treatment Curr Opin Oncol 2010 22 2 138 42 10.1097/CCO.0b013e328335a755 20019613

[B3] Rodriguez-Creixems M Alcala L Munoz P Cercenado E Vicente T Bouza E Bloodstream infections: evolution and trends in the microbiology workload, incidence, and etiology, 1985-2006 Medicine (Baltimore) 2008 87 4 234 49 10.1097/MD.0b013e318182119b 18626306

[B4] Niimi M Firth NA Cannon RD Antifungal drug resistance of oral fungi Odontology 2010 98 1 15 25 10.1007/s10266-009-0118-3 20155503

[B5] Oura M Sternberg TH Wright ET A new antifungal antibiotic, amphotericin B Antibiot Annu 1955 3 566 73 13355328

[B6] Baginski M Sternal K Czub J Borowski E Molecular modelling of membrane activity of amphotericin B, a polyene macrolide antifungal antibiotic Acta Biochim Pol 2005 52 3 655 8 16086075

[B7] Sangalli-Leite F Scorzoni L Mesa-Arango AC Casas C Herrero E Soares Mendes Gianinni MJ Rodriguez-Tudela JL Cuenca-Estrella M Zaragoza O Amphotericin B mediates killing in Cryptococcus neoformans through the induction of a strong oxidative burst Microbes Infect 2011 13 5 457 67 10.1016/j.micinf.2011.01.015 21310262

[B8] Krcmery V Barnes AJ Non-albicans Candida spp. causing fungaemia: pathogenicity and antifungal resistance J Hosp Infect 2002 50 4 243 60 10.1053/jhin.2001.1151 12014897

[B9] Kelly SL Lamb DC Kelly DE Manning NJ Loeffler J Hebart H Schumacher U Einsele H Resistance to fluconazole and cross-resistance to amphotericin B in Candida albicans from AIDS patients caused by defective sterol delta5,6-desaturation FEBS Lett 1997 400 1 80 2 10.1016/S0014-5793(96)01360-9 9000517

[B10] Barker KS Crisp S Wiederhold N Lewis RE Bareither B Eckstein J Barbuch R Bard M Rogers PD Genome-wide expression profiling reveals genes associated with amphotericin B and fluconazole resistance in experimentally induced antifungal resistant isolates of Candida albicans J Antimicrob Chemother 2004 54 2 376 85 10.1093/jac/dkh336 15201233

[B11] Chamilos G Kontoyiannis DP Update on antifungal drug resistance mechanisms of Aspergillus fumigatus Drug Resist Updat 2005 8 6 344 58 10.1016/j.drup.2006.01.001 16488654

[B12] Safdar A Ma J Saliba F Dupont B Wingard JR Hachem RY Mattiuzzi GN Chandrasekar PH Kontoyiannis DP Rolston KV Walsh TJ Champlin RE Raad II Drug-induced nephrotoxicity caused by amphotericin B lipid complex and liposomal amphotericin B: a review and meta-analysis Medicine (Baltimore) 2010 89 4 236 44 10.1097/MD.0b013e3181e9441b 20616663

[B13] Thornton SJ Wasan KM The reformulation of amphotericin B for oral administration to treat systemic fungal infections and visceral leishmaniasis Expert Opin Drug Deliv 2009 6 3 271 84 10.1517/17425240902802861 19327044

[B14] Wasan KM Wasan EK Gershkovich P Zhu X Tidwell RR Werbovetz KA Clement JG Thornton SJ Highly effective oral amphotericin B formulation against murine visceral leishmaniasis J Infect Dis 2009 200 3 357 60 10.1086/600105 19545212

[B15] Wasan EK Gershkovich P Zhao J Zhu X Werbovetz K Tidwell RR Clement JG Thornton SJ Wasan KM A novel tropically stable oral amphotericin B formulation (iCo-010) exhibits efficacy against visceral Leishmaniasis in a murine model PLoS Negl Trop Dis 2010 4 12 e913 10.1371/journal.pntd.0000913 21151883 PMC2998436

[B16] Wasan EK Bartlett K Gershkovich P Sivak O Banno B Wong Z Gagnon J Gates B Leon CG Wasan KM Development and characterization of oral lipid-based amphotericin B formulations with enhanced drug solubility, stability and antifungal activity in rats infected with Aspergillus fumigatus or Candida albicans Int J Pharm 2009 372 1-2 76 84 10.1016/j.ijpharm.2009.01.003 19236839

[B17] Razonable RR Henault M Lee LN Laethem C Johnston PA Watson HL Paya CV Secretion of proinflammatory cytokines and chemokines during amphotericin B exposure is mediated by coactivation of toll-like receptors 1 and 2 Antimicrob Agents Chemother 2005 49 4 1617 21 10.1128/AAC.49.4.1617-1621.2005 15793154 PMC1068636

[B18] Atkinson AJ Jr Bennett JE Amphotericin B pharmacokinetics in humans Antimicrob Agents Chemother 1978 13 2 271 6 646348 10.1128/aac.13.2.271 PMC352226

[B19] Sesana AM Monti-Rocha R Vinhas SA Morais CG Dietze R Lemos EM In vitro activity of amphotericin B cochleates against Leishmania chagasi Mem Inst Oswaldo Cruz 2011 106 2 251 3 10.1590/S0074-02762011000200022 21537689

[B20] Rex JH Pfaller MA Galgiani JN Bartlett MS Espinel-Ingroff A Ghannoum MA Lancaster M Odds FC Rinaldi MG Walsh TJ Barry AL Development of interpretive breakpoints for antifungal susceptibility testing: conceptual framework and analysis of in vitro-in vivo correlation data for fluconazole, itraconazole, and candida infections. Subcommittee on Antifungal Susceptibility Testing of the National Committee for Clinical Laboratory Standards Clin Infect Dis 1997 24 2 235 47 10.1093/clinids/24.2.235 9114154

[B21] Rodriguez-Tudela JL Martinez-Suarez JV Improved medium for fluconazole susceptibility testing of Candida albicans Antimicrob Agents Chemother 1994 38 1 45 8 8141578 10.1128/aac.38.1.45 PMC284394

[B22] Chang HC Chang JJ Chan SH Huang AH Wu TL Lin MC Chang TC Evaluation of Etest for direct antifungal susceptibility testing of yeasts in positive blood cultures J Clin Microbiol 2001 39 4 1328 33 10.1128/JCM.39.4.1328-1333.2001 11283051 PMC87934

[B23] Zager RA Polyene antibiotics: relative degrees of in vitro cytotoxicity and potential effects on tubule phospholipid and ceramide content Am J Kidney Dis 2000 36 2 238 49 10.1053/ajkd.2000.8967 10922301

[B24] Anaissie EJ Paetznick VL Ensign LG Espinel-Ingroff A Galgiani JN Hitchcock CA LaRocco M Patterson T Pfaller MA Rex JH Rinaldi MG Microdilution antifungal susceptibility testing of Candida albicans and Cryptococcus neoformans with and without agitation: an eight-center collaborative study Antimicrob Agents Chemother 1996 40 10 2387 91 8891149 10.1128/aac.40.10.2387 PMC163539

